# Chilean Adaptation and Validation of the Early Adolescent Temperament Questionnaire-Revised Version

**DOI:** 10.3389/fpsyg.2017.02131

**Published:** 2017-12-18

**Authors:** Marianela Hoffmann, J. Carola Pérez, Catalina García, Graciela Rojas, Vania Martínez

**Affiliations:** ^1^Facultad de Psicología, Universidad del Desarrollo, Santiago, Chile; ^2^Escuela de Psicología, Pontificia Universidad Católica de Chile, Santiago, Chile; ^3^Centro de Apego y Regulación Emocional, Facultad de Psicología, Universidad del Desarrollo, Santiago, Chile; ^4^Dirección de Inclusión, Vicerrectoría Académica, Pontificia Universidad Católica de Chile, Santiago, Chile; ^5^Departamento de Psiquiatría y Salud Mental, Hospital Clínico, Facultad de Medicina, Universidad de Chile, Santiago, Chile; ^6^CEMERA, Facultad de Medicina, Universidad de Chile, Santiago, Chile

**Keywords:** temperament, adolescence, adjustment, psychometrics, adaptation, validation

## Abstract

The aim of this study was to develop an adapted version of the Early Adolescent Temperament Questionnaire-Revised (EATQ-R) that would be valid and reliable for assessing temperament and its components in Chileans between 12 and 18 years of age. Originally, Ellis and Rothbart (2001) developed this questionnaire (EATQ-R) to be used in North American adolescents. For the study in Chile, a translation protocol was developed, to maintain the original instrument's cultural and linguistic equivalence in the adapted version. Psychometric properties of the EATQ-R, such as factor structure, internal consistency, and convergent validity, were also assessed. The adaption and validation was carried out in two stages, with two different studies. The first study, which included 612 adolescent students from educational establishments in the cities of Santiago and Concepcion, Chile, developed the Chilean version of the 83-item EATQ-R, which has 13 dimensions, belonging to 4 theoretical factors with adequate internal consistency (Cronbach's alpha = 0.79–0.82). The second study assessed the questionnaire's convergent validity, through its application to 973 adolescent students in Santiago. Results show that the effortful control subscale was significantly inversely related to indicators of adolescent maladjustment, such as substance abuse and behavioral problems. In addition, it was directly associated with indicators of self-concept, including self-esteem and self-efficacy. The opposite pattern was observed when considering negative affect. These findings coincide with current knowledge on the relationship between temperament and adjustment in adolescents.

## Introduction

Historically, differences between children and adolescents have been partly explained in terms of their temperamental characteristics. Although definitions of temperament vary, depending on the theoretical framework utilized, there is consensus on some of its characteristics, such as: (1) it manifests itself from childhood onwards, (2) it has a neurobiological basis, (3) it is consistent over time, (4) and it is multidimensional in nature, encompassing several dimensions of behavior that influence personality development (De Pauw and Mervielde, 2010).

Specifically, the psychobiological model developed by Rothbart and her collaborators conceptualizes temperament as individual constitutional differences in reactivity and as the self-regulation of affect, activities, and attention-related processes (Rothbart and Bates, 2006; Rothbart, 2011). Constitutional aspects refer to the relative durability of biological influences on individuals, which is modeled by inheritance, maturation, and experience (Rothbart and Bates, 2006). On the other hand, reactivity concerns the physiological excitability of neural systems, while self-regulation refers to processes that allow us to modulate this automatic and involuntary reactivity (Rothbart, 2011).

Self-regulation includes the ability to direct one's attention, inhibitory processes, and behavioral activation. Regulatory attention is the capacity to focus and redirect one's attention when necessary. Behavioral inhibition processes make it possible to suppress a dominant response when required, while behavioral activation processes let us initiate and sustain a non-dominant response (Rothbart and Rueda, 2005).

Factor analyses of Rothbart's instruments have provided evidence that temperament structure in all age groups can be mostly explained by three dimensions, which have been labeled as effortful control (which represents self-regulation processes), surgency, and negative affect (the latter two both represent reactivity processes) (Rothbart and Bates, 2006). In the case of early adolescence a fourth factor, called affiliativeness, has become evident (Putnam et al., 2001).

Effortful control is the ability to suppress a dominant response and produce a subdominant one instead, which denotes a self-regulatory mechanism. Surgency refers to the level of activity and tendency to approach novel situations, and negative affect represents proneness to experiencing negative emotions such as discomfort, fear, dysphoria, anger, and/or frustration (Rothbart and Bates, 2006). Lastly, affiliativeness involves concern for others and a desire for closeness with others (Ellis and Rothbart, 2001; Putnam et al., 2001).

Temperament has been extensively studied in children, and several forms of measurement are available, such as surveys for parents and other adults, and observational instruments for experimental settings (Rothbart and Bates, 2006). Adolescent temperament is usually measured via self-report instruments and parental report scales. Among these, the Early Adolescent Temperament Questionnaire - Revised (EATQ-R), developed by Ellis and Rothbart (2001), is one of the leading instruments for measuring adolescent temperament.

This article presents the validation of the EATQ-R in Chile. This study involved two successive stages: first, the questionnaire was translated and adapted into Spanish and its psychometric properties were analyzed (Study 1); then, the concurrent validity of the instrument was determined (Study 2). The Early Childhood Behavior Questionnaire (ECBQ) was previously adapted and validated by Bowen et al. (2004) to measure temperament in Chilean infant children (16–35 months), and the current adaption and validation of EATQ-R will provide a culturally appropriate instrument to evaluate temperament in Chilean adolescents; together, these questionnaires allow us monitor temperament of Chileans throughout the life cycle.

The relevance of assessing temperament is widely accepted, as it has been shown to play a fundamental role in the socio-emotional adjustment of children and adolescents (Coplan and Bullock, 2012; Al-Hendawi, 2013; Valiente et al., 2013), especially in the development of psychopathology (Griffith et al., 2010). According to studies on temperament and psychopathology, children and adolescents who are more vulnerable to displaying internalizing and externalizing symptomatology tend to have poorer self-regulation skills (Baetens et al., 2011; Muris et al., 2011).

Vulnerability to internalizing psychopathology is characterized by a combination of high levels of negative emotionality and low levels of effortful control (Calkins et al., 2016). More specifically, high levels of negative emotionality may increase adolescents' propensity to develop psychological disorders (Forbes et al., 2017); nevertheless, the negative impact of this emotional reactivity may be buffered by effortful control (Muris and Ollendick, 2005). For instance, a stressful life event may trigger negative emotions in adolescents, especially in those characterized by high emotional reactivity levels, but only adolescents who display low levels of effortful control are likely to have problems effectively managing these negative feelings and may, therefore, react through internalizing symptomatology (Muris and Ollendick, 2005). In contrast, adolescents with high levels of effortful control can regulate their emotional states by using more flexible and effective strategies (Lengua and Long, 2002), such as redirecting their attention (Forbes et al., 2017), which reduces their risk of developing mental health problems.

The relationship between temperamental traits and psychopathology is visible not only in adolescents' internalizing symptomatology, but also in their externalizing symptomatology, for example, substance abuse and attention deficit hyperactivity disorder, among others (Martel et al., 2009; Baetens et al., 2011; Brumley and Jaffee, 2016).

Additionally, temperamental dimensions have being related with adolescent adjustment indicators, such as self-esteem (Heinonen et al., 2002; Robins et al., 2010), self-efficacy (Findley, 2013), and pro-social behaviors (Hirvonen et al., 2017).

Robins et al. (2010) found that Mexican-American adolescents with high self-esteem showed higher levels of effortful control and lower levels of aggression. However, no significant relations were found between self-esteem and negative affectivity (including fear and frustration). Heinonen et al. (2002) found that mother's perceptions of adolescent's temperament as “difficult” at ages 12 and 15 predicted adolescents' low self-esteem in late adolescence. Additionally, Watson et al. (2002) reported that self-esteem is strongly negatively correlated with neuroticism and negative affectivity.

Findley (2013) reported that self-concept clarity and self-esteem were negatively related to negative affect and fearful temperament, in a sample of early and middle adolescent students from an ethnically diverse school.

Surgency has been associated with lower levels of internalizing symptoms (Oldehinkel et al., 2004), for example with less anxiety (Snyder et al., 2015), and with higher levels of externalizing symptoms (Muris et al., 2011).

Finally, affiliativeness has been associated with higher depressive symptoms (Hankin et al., 2010), but with lower levels of anxiety. It has also been linked to more antisocial behavior and greater victimization by peers (Snyder et al., 2015).

In the present study, we hypothesize that:

- Taking in consideration the adaptation efforts conducted in other countries (Chang, 2004; Muris and Meesters, 2009; Viñas et al., 2015), we expect that we will need to modify some items and eliminate others, with poor psychometric properties. Despite this, we predict that we find the four factors inherent to the original instrument (i.e., Effortful Control, Surgency, Affiliativeness, and Negative Affect) (Ellis and Rothbart, 2001).
- Effortful control will be directly associated with indicators of self-concept, including self-esteem and self-efficacy. In addition, effortful control will be significantly inversely related to adolescent behavioral problems and/or adolescent substance abuse. On the other hand, the presence of these problems will be directly associated with negative affect, which will also be inversely related to indicators of adolescent self-concept. Finally, since Affiliativeness and Surgency have been found to have apparently contradictory associations with indicators of adolescent adjustment (Nigg, 2006), we do not expect to find a specific pattern of associations between these temperamental dimensions and self-concept and mal-adjustment indicators.

## Study 1

### Material and methods

#### Participants

The convenience sample comprised 612 adolescents (from 7 to 12th grade) attending seven educational institutions located in the Chilean cities of Santiago (3 schools: 67.2% of participants) and Concepción (4 schools: 32.8% of participants). The average age of the participants was 14.96 years (SD = 1.76), and 49.7% were female (see Table 1). In terms of the type of school, 42.4% of the participants attended municipal (public) schools, 29.1% went to subsidized private schools, and 28.5% attended private schools. With respect to their living arrangement, 65.9% of the adolescents lived with both of their parents, 19.6% with one parent (mother or father), and 8.1% with one parent and that parent's new partner. Only 0.6% of the participants did not live with either parent.

**Table 1:** Sample distribution by Gender, Course, and City of Residence.

	**School grades**						**Total**
	**Primary school**		**Secondary school**				
	**7th Grade (%)**	**8th Grade (%)**	**9th Grade (%)**	**10th Grade (%)**	**11th Grade (%)**	**12th Grade (%)**	
**SANTIAGO**							
Female	43.3	46.4	53.4	51.7	49.3	57.4	50.1% *N* = 204
Male	56.7	53.6	46.6	48.3	50.7	42.6	49.9% *N* = 203
**CONCEPCIÓN**							
Female	58.3	50.0	67.9	35.0	40.0	42.1	49.8% *N* = 102
Male	41.7	50.0	32.1	65.0	60.0	57.9	50.2% *N* = 103
Total	100	100	100	100	100	100	*N* = 612
	*N* = 120	*N* = 97	*N* = 101	*N* = 127	*N* = 101	*N* = 66	

#### Ethics statement

The Ethics Committee of Universidad del Desarrollo, Chile reviewed and approved the study. Students' parents were asked to authorize their children to participate by signing an informed consent form, and then adolescents who received their parents' authorization and who agreed to participate in the study voluntarily and anonymously expressed their decision by signing an informed assent form.

#### Instrument

The Early Adolescent Temperament Questionnaire—Revised (EATQ-R) measures temperament in adolescents. The instrument was originally created to be administered to adolescents between 9 and 15 years old. The scale includes 103 items, divided into 13 dimensions that reflect 4 factors: (1) Affiliativeness, which includes *affiliation* (desiring warmth and closeness with others), *pleasure sensitivity* (pleasure associated with activities and stimuli that involve low intensity, low complexity, and low novelty), and *perceptual sensitivity* (detection of low-intensity stimulation in one's environment). (2) Effortful Control, which includes *attention* (the ability to focus one's attention and shift focus at will), *activation control* (ability to perform an action despite a strong tendency to avoid it), and *inhibitory control* (ability to plan and suppress inappropriate responses). (3) Negative Affect, which includes *frustration*, due to the interruption of an ongoing task or due to being prevented from achieving a goal; physical and verbal *aggression*, exerted both directly and indirectly; and *depressive mood*. (4) *Surgency*, which includes low *fear* and *shyness* (high behavioral inhibition when faced with novelty and change, especially in the social sphere) and *high intensity pleasure* (intense pleasure derived from high-intensity or high-novelty activities) (Ellis, 2002). Lastly, the *activity level* dimension refers to physical participation in activities requiring high levels of physical activity. This dimension is not part of the other four higher order temperament factors (Ellis and Rothbart, 2001).

Each item was answered using a 5-point Likert scale (1= *Almost always untrue of you*; 5= *Almost always true of you*). Total scores per subscale were calculated by adding up scores of the corresponding items, with high scores reflecting the presence of a given temperamental characteristic. The reliability indexes (Cronbach's alpha) reported for the dimensions ranged from 0.64 to 0.82 (Ellis and Rothbart, 2001; Ellis, 2002).

#### Procedures

##### Translation

In order to adapt this instrument to the Chilean context, the original EATQ-R in English and a Spanish version (translated in Spain) were taken into account. The latter is the official translation provided by the authors for Spanish speakers^1^. Two translators (a man and a woman) who were native Spanish-speakers translated the original English instrument into Spanish. Each of them translated the instrument from English into Chilean Spanish independently, using the official Spanish version as a frame of reference for their own translations.

Afterwards, the research team merged both translations, which resulted in a pilot version that preserved the meaning of the original questionnaire, but whose textual form reflected local culture and the Spanish language as used in Chile. To generate this final product, the researchers considered the original Spanish version from Spain, both translations, and theoretical information about the scales and items of the original instrument. This process yielded 52 items that remained unchanged from the official Spanish version of the EATQ-R and 51 items that underwent textual modifications. For instance, the vocabulary used to refer to emotional states was modified to match local reality more closely (“me enfado …” was replaced by “me enojo…”; “me siento retraído” was replaced by “me siento avergonzado”); and the sports mentioned were replaced by others which are more commonly practiced in the Chile, while preserving the type of sport (i.e., “…como el esquí acuático” was replaced by “… como el esquí acuático y motocross”).

##### Administration

Seven educational institutions of different types, located in Santiago and Concepción, were asked to participate in the study. Authorized adolescents completed the questionnaire in their classroom.

##### Cognitive interview

The pilot version of the questionnaire was administered to 18 students through individual interviews. The pilot sample was comprised of one boy and one girl from each age group (12–14, 15–16, and 17–18 years) in each type of school (municipal, subsidized private, and private). The adolescents answered the self-report questionnaire in a room with a research assistant, who asked questions about their understanding of the items after they completed them. After completing 20 items, the students were asked to state their opinion about the clarity of the questions, the instructions provided, and their way of answering them. After answering the whole questionnaire, they were asked to comment on its length, its general degree of clarity, its difficulty, and on whether or not it was enjoyable to answer. The assistant kept a comprehensive record of the students' comments. Based on this pilot result, 14 items were modified (six items that had been carried over from the original Spanish version and eight items that had been modified for Chile). In addition, two of these items were modified to avoid using double negation, which can negatively impact comprehension.

##### Data analysis

To begin, we estimated the measurement model specified by the authors (Ellis and Rothbart, 2001), which includes 13 factors (11 temperamental dimensions and 2 behavioral scales), allowing the factors to be correlated with one another, using Confirmatory Factor Analysis (CFA). Since the levels of fit of this first model were inadequate, two additional successive CFAs were performed, preserving the original factor structure and removing items with a low factor loading (≤0.30) in the national sample. These models were estimated by setting the variance of the factors at 1 using the MLM estimation algorithm (MPlus-6), an estimator that is less sensitive to the violation of the multivariate normality assumption (Muthén and Muthén, 1998-2010; Institute for Digital Research and Education, UCLA)^2^. We present the standardized factor loadings (STDY, see Table 2), as well as the fit indicators for the initial and the Chilean final model.

**Table 2:** Factor loading pattern of EATQ-R scales.

**Subscales**	**EATQ-R (Chilean version 1)**		**EATQ-R (Chilean final version)**	
	**Items**	**Range**	**Items**	**Range**
**EFFORTFUL CONTROL**				
Activation control	7 (R); 24(R); 32; **55(R)**; 63; 65; 66; 82(R)	0.13–0.65	7 (R); 32; 63; 65; 66; 82 (R)	0.38–0.67
Inhibitory control	12(R); 19; 20; 21(R); 22(R); 39(R); **45(R)**, 46(R); 71; 81; 89	0.01–0.55	19; 20; 46(R); 81; 89	0.31–0.64
Attention	25; 44(R); 56(R); 62(R); **67**; 86(R); 97	0.22–0.63	25; 44(R); 56(R); 62(R); 86(R); 97	0.33–0.64
**AFFILIATIVENESS**				
Affiliation	23; 38; 47; 51; 75; 78; 88; 94	0.39–0.55	23; 38; 47; 51; 75; 78; 88; 94	0.34–0.54
Pleasure sensitivity	28; 30; 34; 48; 50; 70; 80	0.27–0.75	28; 30; 48; 50; 70; 80	0.40–0.77
Perceptual sensitivity	4; 11; 31; 35; 41; **92(R)**	0.16–0.74	4; 11; 31; 35	0.47–0.76
**NEGATIVE AFFECT**				
Frustration	40(R); 42; 58; 73; 79; 91; 98; 101; 102	0.38–0.62	40(R); 42; 58; 73; 79; 91; 98; 101; 102	0.38–0.61
Aggression	5; 10; 18; 33; 37; 59; 68; 72; **74(R)**; 84; 96	0.20–0.63	5; 10; 18; 33; 37; 59; 72; 84; 96	0.36–0.65
Depressive mood	13; 15; 26(R); 49; 60; 90	0.42–0.76	13; 15; 26(R); 49; 60; 90	0.42–0.76
**SURGENCY**				
Shyness	6; 9; 43; 52(R); 69; 76; 87(R)	0.32–0.75	6; 9; 43; 52(R); 69; 76; 87(R)	0.33–0.84
High Intensity pleasure	**2**; **8**; 14(R); 17(R); **27**; **29**; 53; 61; 95; 99; 103	0.11–0.69	14(R); 17(R); 53; 61; 95; 99; 103	0.33–0.68
Fear	3; 54; 57; 77; 85; 93	0.29–0.45	3; 57; 77; 85; 93	0.32–0.43
Activity Level	1; 16; 36; 64; 83; 100	0.26–0.77	1; 36; 64; 83; 100	0.46–0.76

*Magnitude of factorial loadings (independent of the sign). Bold item numbers have factorial loading <0.30*.

To measure the fit of the estimated CFA, several indicators were used: (a) Satorra-Bentler goodness of fit index (*SB*χ*2*)—when values are non-significant (*p* ≥ 0.05), it indicates that the model fits the data well (Satorra and Bentler, 1994); (b) Root Mean Square Error of Approximation (*RMSEA*), in which values ≤0.05 indicate good fit, while values over 0.05 and under 0.08 indicate acceptable fit (Browne and Cudeck, 1993), though other authors indicate that a cut-off of 0.06 is more adequate (Hu and Bentler, 1999); (c) Standardized Root-Mean-Square Residual (*SRMR*), in which values < 0.05 indicate a good model fit (Byrne, 1998; Diamantopoulos and Siguaw, 2000), although values ≤0.08 are considered to be acceptable (Hu and Bentler, 1999); and (d) Comparative Fit Index (*CFI*) (Bentler, 1990), for which Hu and Bentler (1999) suggest that *CFI* values ≥ 0.95 indicate a good model fit.

In the second phase, Exploratory Factor Analysis (EFA) was conducted over 13 level-1 factors of temperamental dimensions (Ellis and Rothbart, 2001; Ellis, 2002). The number of factors was determined using a scree-test, and the factorial solution was estimated by the method of principal axes (SPSS 15.0) with an Oblim rotation. Lastly, we estimated the internal consistency of each factor and their subscales using Cronbach's alpha.

### Results

#### Factor structure of the scale

In the initial factorial solution (Chilean version 1), results showed that all items included within the factors of Affiliation (factor loadings range: 0.4–0.6, 8 items), Pleasure Sensitivity (0.3–0.8, 7 items), Frustration (0.4–0.6, 9 items), Depressive Mood (0.4–0.8, 6 items), Shyness (0.3–0.8, 7 items), and Activity Level (0.3–0.8, 6 items) displayed significant loadings and were adequate in magnitude.

Though Fear behaved similarly, its factor loadings were lower (range 0.3–0.5, 6 items). In the case of the Activation Control factor, seven of the items displayed significant factor loadings, ranging from 0.3 to 0.7, except for item 55, which had non-significant loadings for this factor.

In the case of Inhibitory Control, item 45 had non- significant loadings for this factor; in contrast, its other items displayed significant factor loadings, ranging from 0.3 to 0.6 (9 items). Item 67 in Attention, item 74 in Aggression, and item 92 in Perceptual Sensitivity also behaved similarly. In all cases, items displayed low but significant factor loadings (below 0.3). The remaining items displayed loadings—in their respective factors—that ranged between 0.3 and 0.6 in Attention (6 items), from 0.3 to 0.7 in Perceptual Sensitivity (5 items), and from 0.3 to 0.6 in Aggression (10 items).

The High Intensity Pleasure factor had the largest number of problematic items; specifically, items 2, 8, 27, and 29 displayed factor loadings which were lower than 0.3 (but still significant). Its other seven items displayed loadings ranging from 0.3 to 0.7 (see Table 2). This model showed the following fit indexes values: (a) *SB*χ*2* = 8781.71 (5071 *df*.), *p* < 0.001; (b) *RMSEA* = 0.039 (90% CI 0.038–0.041); (c) *SRMR* = 0.071; (d) *CFI* = 0.66.

In the final factorial solution (Chilean final Version), the Activation Control factor had 6 items (loading factor range: 0.4–0.7), Inhibitory Control had 5 items (range: 0.3–0.6), Attention had 6 items (range: 0.3–0.6), Affiliation had 8 items (range: 0.3–0.5), Pleasure Sensitivity had 6 items (range: 0.4–0.8), Perceptual Sensitivity had 4 items (range: 0.5–0.8), Frustration had 9 items (range: 0.4–0.6), Aggression had 9 items (range: 0.4–0.7), Depressive Mood had 6 items (range: 0.4–0.8), Shyness had 7 items (range: 0.3–0.8), High Intensity Pleasure had 7 items (range: 0.3–0.7), Fear had 5 items (range: 0.3–0.4), and Activity Level had 5 items (range 0.5–0.8) (additional information in Appendix 1). Additionally, the errors of items 14 and 17 were correlated (*r* = 0.62, *p* ≤ 0.001). The fit index values of the final factorial solution were: (a) *SB*χ*2* = 5705.84 (3241 *df*.), *p* < 0.001; (b) *RMSEA* = 0.039 (90% CI 0.037–0.041); (c) *SRMR* = 0.068; (d) *CFI* = 0.74.

Table 3 presents the final correlations estimated by the model. Several factors correlated with each other, although there were some exceptions. The most important (*r*_s_ ≥ 0.30) correlations were: A positive relation between inhibitory control and activation control (*r* = 0.44); and positive correlations between attention with inhibitory control (*r* = 0.40); and with activation control (*r* = 0.44). All these scales correspond to the Effortful Control factor. Additionally, inhibitory control was negatively associated with aggression (*r* = −0.44), and a negative relation between attention and aggression (*r* = −0.32) was found. Frustration was positively related to aggression (*r* = 0.46) and depressive mood (*r* = 0.38). These last three scales correspond to the Negative Affect factor. In terms of the dimensions of Affiliativeness, affiliation was positively related to pleasure sensitivity (*r* = 0.39), and pleasure sensitivity was related to perceptual sensitivity (*r* = 0.33). Finally, high intensity pleasure was positively related to activity level (*r* = 0.45) and negatively related to shyness (*r* = −0.31).

**Table 3:** Correlations.

**Dimensions**	**2**	**3**	**4**	**5**	**6**	**7**	**8**	**9**	**10**	**11**	**12**	**13**
1. Activation control	0.44^***^	0.44^***^	0.12^**^	0.16^***^	0.15^***^	−0.12^**^	−0.27^***^	−0.09^*^	−0.04	0.02	0.12^**^	0.17^***^
2. Inhibitory control		0.40^***^	0.13^**^	0.19^***^	0.13^***^	−0.29^***^	−0.44^***^	−0.18^***^	−0.05	0.05	0.10^*^	0.24^***^
3. Attention			−0.03	0.09^*^	−0.04	−0.25^***^	−0.32^***^	−0.29^***^	−0.14^***^	0.08	−0.05	0.08
4. Affiliation				0.39^***^	0.22^***^	0.12^**^	−0.03	0.16^***^	−0.17^***^	0.11^**^	0.24^***^	0.18^***^
5. Pleasure sensitivity					0.33^***^	0.01	−0.12^**^	0.11^**^	−0.04	−0.07	−0.19^***^	0.15^***^
6. Perceptual sensitivity						0.18^***^	−0.02	0.16^***^	−0.10	0.04	0.13^**^	0.09^*^
7. Frustration							0.46^***^	0.38^***^	0.08^*^	0.02	0.18^***^	−0.05
8. Aggression								0.19^***^	−0.04	0.26^***^	−0.13^***^	0.08^*^
9. Depressive mood									0.29^***^	−0.21^***^	0.27^***^	−0.25^***^
10. Shyness										−0.31^***^	0.22^***^	−0.26^***^
11. High intensity pleasure											−0.23^***^	0.45^***^
12. Fear												−0.08
13. Activity level												

*Bolded numbers are mentioned in the text*.

*** *p < 0.001*;

** *p < 0.01*;

* *p < 0.05*.

The EFA indicated a four-factor solution (see Table 4). The first factor was Effortful Control, which included Attention, Activation Control, and Inhibitory Control dimensions. The second factor is Affiliativeness, with the dimensions of Affiliation, Pleasure Sensitivity, and Perceptual Sensitivity dimensions. Both of these factors replicated the original questionnaire's factor structure. Surgency, the third factor, included three original dimensions (Shyness, High Intensity Pleasure, and Fear) and also includes the Activity Level dimension. Finally, Frustration and Aggression were included in the fourth factor, Negative Affect factor. This factor also includes Depressive Mood, which had an additional significant factor loading in Surgency (a negative factor loading).

**Table 4:** Dimensions of EATQ-R.

**Dimensions**	**Factors**			
	**1**	**2**	**3**	**4**
Activation control	**0.76**			
Inhibitory control	**0.53**			
Attention	**0.58**			
Affiliation		**0.67**		
Pleasure sensitivity		**0.62**		
Perceptual sensitivity		**0.40**		
Frustration				**0.66**
Aggression				**0.70**
Depressive mood			−0.43	**0.35**
Shyness			**−0.50**	
High intensity pleasure			**0.69**	
Fear		0.34	**−0.37**	
Activity level			**0.56**	

*Factor loading <0.30 were eliminated. Principal Axis Factoring and Oblimin Rotation. Factor correlations are: factor 1 with factor 2 r = 0.25; factor 1 with factor 3 r = 0.10; factor 1 with factor 4 r = −0.34; factor 2 with factor 3 r = −0.03; factor 2 with factor 4 r = 0.18; factor 3 with factor 4 r = −0.09. Bolded dimensions belong to respective factors*.

#### Reliability analysis

The reliability of the EATQ-R dimensions ranged from 0.44 to 0.77. Reliability problems were found in Activation Control (0.68), Inhibitory Control (0.58), Attention (0.66), and Affiliation (0.67). The dimensions of Pleasure Sensitivity, Perceptual Sensitivity, each dimension of Negative Affect, Shyness, High Intensity Pleasure, and Activity Level displayed reliability levels equal to or higher than 0.70. Fear was the least reliable scale. Reliability displayed by the four factors was adequate, ranging from 0.79 (Affiliativeness) to 0.82 (Negative Affect, see Table 5).

**Table 5:** Reliability.

**Factor**	**Dimension**	**Number of items**	**Crobanch alpha**
**Effortful control**		**17**	**0.80**
	Activation control	6	0.68
	Inhibitory control	5	0.58
	Attention	6	0.66
**Affiliativeness**		**18**	**0.79**
	Affiliation	8	0.67
	Pleasure sensitivity	6	0.76
	Perceptual sensitivity	4	0.70
**Negative Affect**		**24**	**0.82**
	Frustration	9	0.72
	Aggression	9	0.72
	Depressive mood	6	0.73
**Surgency**^a^		**24**	**0.81**
	Shyness	7	0.77
	High intensity pleasure	7	0.71
	Fear	5	0.44
	Activity level	5	0.77

a *All items were recoded so that the highest score in the highest level of Surgency*.

### Discussion

The present study describes the adaptation and validation of the EATQ-R in Chilean adolescents between 12 and 18 years of age. CFA made it possible to determine whether the items translated into Spanish had item loadings for the same 13 dimensions proposed in the original version of the instrument. As initial results showed inadequate adjustment levels, new CFAs were conducted, which preserved the structure of the factors proposed theoretically by Rothbart but eliminated 20 items with low factor loadings (<0.3). The final version of the instrument comprises 83 items that displayed adequate and significant factor loadings.

In this final version, there were inconsistencies between fit indexes. The *RMSEA* (0.039) and *SRMR* (0.068)—both categorized as absolute fit indicators—indicated an adequate level of model fit; in contrast, the *SB*χ*2* and *CFI* indexes revealed a poor model fit. This same inconsistency was reported by Kim et al. (2003), who conducted a CFA with six factors of the EATQ-R using individual items as indicators (a strategy comparable to that used in the present study). A similar inconsistency was also reported in adaptation studies carried out by Viñas et al. (2015) and Muris and Meesters (2009).

Selecting and reporting multiple indicators is a common and usually recommended practice. This is because no single indicator can assess the fit of the models contrasted under the multiple conditions involved in their estimation, such as sample size, estimation methods, and the relationship among the indicators in each factor (Fan et al., 1999). Multiple simulation studies have been conducted to assess how different indexes perform when these conditions vary (Fan et al., 1999; Jackson, 2001). Systematically, it has been found that the *RMSEA* and *SRMR* are affected by sample size—the larger the size, the better the fit (Jackson, 2001). However, *RMSEA* is a recommended indicator, as it is sensitive to poor model specification (Fan et al., 1999). On the other hand, even though *CFI* is more independent of sample size, it is affected by the size of factor loadings, the estimation method used (with greater values being obtained with Generalized Least Squares than with Maximum Likelihood), and its interactions, among other aspects (Ding et al., 1995).

With regard to the Chilean model indicators that showed a poor fit, *SB*χ*2* is not appropriate for large sample sizes (Kenny, 2012) and *CFI* is not informative when the “*RMSEA* for the null model is <0.158” (Kenny, 2012), which is the case of the presented model (our null model *RMSEA* is 0.08). Additionally, Raykov (2000, 2005) argues that *CFI* is a biased measure. Thus, based on the adequate absolute fit indices, we consider this model to be acceptable.

With regard to level-2 factors, the final Spanish version replicates the four factors of the original model, which includes Effortful Control, Affiliativeness, Surgency, and Negative Affect. In contrast to the original EATQ-R solution (Ellis, 2002), our results include Activity Level within the Surgency factor. In addition, the Depressive Mood dimension had significant loadings in two factors: Negative Affect and Surgency. Ellis and Rothbart (2001) developed Depressive Mood as an additional behavioral scale, therefore its inclusion as a temperament dimension warrants further study.

Considering the four theoretical factors that comprise the EATQ-R questionnaire (Affiliativeness, Effortful Control, Negative Affect, and Surgency), their internal consistency was adequate (Cronbach's alpha = 0.79–0.82). Moreover, the values were similar to those published by Ellis (2002). Nevertheless, when considering the reliability of the dimensions that comprise these factors, the observed internal consistency was lower. The least reliable dimensions were Fear and Inhibitory Control, which reached lower values than those in the original English version reported by Ellis and Rothbart (2001). Other studies have also reported problems with the reliability of some dimensions. For example, Fear, Inhibitory Control, and Attention displayed a reliability value lower than 0.65 in the adaptation developed by Ellis (2002) and in versions in other languages (Chang, 2004; Muris and Meesters, 2009; Pérez and Cumsille, 2012).

Once the factor structure of the Chilean version of the EATQ-R has been established, its construct validity needs to be further explored. Convergent validity—the extent to which a scale correlates with another or to other measures designed to assess similar constructs—and Discriminant Validity—the extent to which a scale does not correlate with dissimilar measures—are often used to evaluate the construct validity of instruments. In light of this, as previously indicated in the introduction, in the second phase of our study, the convergent validity of Chilean version of EATQ-R, with respect to indicators of socio-emotional adjustment, was explored.

## Study 2

### Materials and methods

#### Participants

The convenience sample consisted of 973 adolescents (from 7 to 11th grade) attending 29 schools located in Santiago. Their average age was 14.41 years (SD = 1.42) and 68.8% were female (see Table 6). In terms of their school, 38.8% of the participants attended municipal (public) schools, 56% attended subsidized private schools, and 5.1% attended a private school. With regard to their living arrangement, 67.3% of the adolescents lived with both of their parents, 24.5% with one parent (mother or father), and 8.1% with one parent and that parent's new partner.

**Table 6:** Sample distribution by Gender and Grade.

	**Primary school**			**Secondary school**		
	**7th Grade (%)**	**8th Grade (%)**	**9th Grade (%)**	**10th Grade (%)**	**11th Grade (%)**	**Total**
Female	55.6	77.4	73.6	72.3	55.1	68.8% *N* = 669
Male	44.4	22.6	26.4	27.7	44.9	31.2% *N* = 304
Total	100	100	100	100	100	100%
	*N* = 133	*N* = 190	*N* = 254	*N* = 249	*N* = 147	*N* = 973

#### Procedures

Once municipal and subsidized private schools provided their authorization for this study, consent was sought from students and their parents. The adolescents who were authorized by their parents, and who agreed to participate (by signing an informed assent form), answered a questionnaire in their classroom (which contained, among others, EATQ-R and scales of adjustment and maladjustment in adolescents). In the case of the participating private schools, recruitment was conducted by contacting the parents directly (snowball sampling), and the adolescents who were authorized completed the questionnaire in their homes. The Ethics Committee of Universidad del Desarrollo, Chile, following the same ethical procedure mentioned in Study 1, reviewed and approved the study.

#### Instruments

##### Chilean adaptation of the early adolescent temperament questionnaire–revised

The scale includes 83-items, divided into 13 dimensions. Each item is answered using a 5-point Likert scale (1= *Almost always untrue of you*; 5= *Almost always true of you*).

In this study, only 71^3^ items were included, due to the extensive length of the other instruments used. The selected factors and dimensions were: (1) Affiliativeness, which includes *affiliation, pleasure sensitivity*, and *perceptual sensitivity*; (2) Effortful Control, which considers the dimensions *attention, activation control*, and *inhibitory control*; (3) Negative Affect, which includes *frustration, aggression*, and *depressive mood*; and (4) Surgency, which includes *fear, shyness*, and *high intensity pleasure*.

##### Scales of adjustment and maladjustment in adolescents

These three scales have been previously used in Chile (Cumsille et al., 2014). (1) *Depressive Symptomatology Scale*, which includes seven items that measure the frequency of the presence of negative affect (Cronbach's alpha = 0.86). Some of these items are: “In the last 30 days, how often have you felt depressed tense, or irritable?” Each item was answered using a 6-point Likert scale (1 = *never*; 6 = *almost every day)*. (2) *Self-Concept Scale*, that includes 11 items that represent adolescent self-assessment. The scale was constructed using items similar to those of Harter's Global Self-Assessment subscale (Harter, 1982) and others related to general self-efficacy expectations, adapted from the Self-Efficacy scale developed by Sherer and Adams (1983). Adolescents are asked to report the degree to which each of the statements describes them on a 5-point Likert scale (1 = *It does not describe me at all*−5 = *It describes me completely*). The scale includes two factors: Self-Esteem and Self-efficacy. The former is comprised of five items (examples: “I feel quite sure of myself,” “I'm happy to be the way I am”), with a 0.86 Cronbach's alpha value. The latter factor includes six items (examples: “I can focus on a task and persevere,” “I can be successful in what I set out to do”), with a Cronbach's alpha estimated at 0.86. (*3) Substance Use and Behavioral Problems Scale*. This scale includes 11 items that assess the frequency of use alcohol and marijuana by adolescents and how often they engaged in problematic behavior during the past 6 months. Each item was answered using a 7-point Likert scale (1 = *Never in my life*; 7 = *More than once per week*). The scale includes two factors: Substance use (examples: “You have smoked cigarettes,” “You have drank alcohol until you got drunk”) and Antisocial behavior (examples: “You have taken something from a store without paying,” “You have become involved in a group/gang fight”). The former includes five items (Cronbach's alpha = 0.80) and the latter includes seven (Cronbach's alpha = 0.66).

### Results

Table 7 presents the correlations between the four temperament factors (Effortful Control, Affiliativeness, Negative Affect, and Surgency) and indicators of adolescents' adjustment and maladjustment, which were consistent with our hypotheses. Effortful Control was significantly and negatively (−0.20 < *r* < −0.33) related with indicators of adolescent maladjustment, considering both depressive and externalizing symptoms. It was also directly correlated with both indicators of self-concept (self-efficacy = *r* < 0.60 and self-esteem = *r* < 0.45). The opposite pattern was observed when considering Negative Affect, which was directly related to the maladjustment scale (0.15 < *r* < 0.58) and inversely related to the adolescent adjustment scales (−0.30 < *r* < −0.45).

**Table 7:** Correlations of adolescent temperament and adolescent adjustment indicators.

**Factors**	**2**	**3**	**4**	**5**	**6**	**7**	**8**	**9**
1. Effortful control	−0.44^***^ (*N* = 965)	0.20^***^ (*N* = 953)	0.07^*^ (*N* = 964)	−0.32^***^ (*N* = 964)	−0.33^***^ (*N* = 963)	−0.20^***^ (*N* = 964)	0.45^***^ (*N* = 965)	0.60^***^ (*N* = 965)
2. Negative affect		0.19^***^ (*N* = 954)	−0.17^***^ (*N* = 967)	0.58^***^ (*N* = 967)	0.24^***^ (*N* = 966)	0.15^***^ (*N* = 967)	−0.45^***^ (*N* = 968)	−0.30^***^ (*N* = 968)
3. Affiliativeness			−0.07^*^ (*N* = 953)	0.11^***^ (*N* = 953)	−0.12^***^ (*N* = 952)	0.02 (*N* = 953)	0.07^*^ (*N* = 954)	0.19^***^ (*N* = 954)
4. Surgency				−0.19^**^ (*N* = 966)	0.25^***^ (*N* = 965)	0.23^***^ (*N* = 966)	0.22^***^ (*N* = 967)	0.19^***^ (*N* = 967)
5. Depressive symptoms					0.18^***^ (*N* = 970)	0.20^***^ (*N* = 971)	−0.60^***^ (*N* = 972)	−0.37^***^ (*N* = 972)
6. Antisocial behavior						0.51^***^ (*N* = 970)	−0.11^***^ (*N* = 971)	−0.14^***^ (*N* = 971)
7. Substance use/abuse							−0.09^**^ (*N* = 972)	−0.08^*^ (*N* = 972)
8. Self-esteem								0.67^***^ (*N* = 973)
9. Self-efficacy								

*** *p < 0.001*;

** *p < 0.01*;

* *p > 0.05*.

The Affiliativeness and Surgency scales displayed a more complex pattern of correlation with adjustment and maladjustment indicators. Moreover, their correlations were lower in magnitude than those established with the dimensions of Effortful Control and Negative Affect dimensions. For example, Surgency was significantly and positively related (0.19 < *r* < 0.22) with indicators of adolescent adjustment, and it was also positively related to antisocial behavior (*r* = 0.25) and substance abuse (*r* = 0.23). Finally, there was a negative correlation between Surgency and depressive symptoms.

Similarly, Affiliativeness was significantly and positively related (0.07 < *r* < 0.19) with indicators of adolescent adjustment, and at the same time, it was positively related with depressive symptomatology *(r* = 0.11). In addition, Affiliativeness was significantly and negatively related with antisocial behavior (*r* = −0.12).

## General discussion

The present study describes the psychometric properties, such as factor structure, internal consistency, and convergent validity, of the EATQ-R questionnaire, when administered to a sample of Chilean adolescents between 12 and 18 years of age.

EATQ-R was adapted by following a translation and re-translation protocol intended to preserve cultural and linguistic equivalence with the original version. Based on factorial analysis, the Chilean version of EATQ-R is composed of only 83 of the original 103 items. It is important to mention that adaptation processes carried out in other countries have also removed items to reach more adequate factor solutions (Chang, 2004; Viñas et al., 2015). In fact, many of the items that caused problems in the Chilean version were eliminated in a new version of the instrument developed by Ellis (2002).

In the second study, correlations were established between temperamental factors assessed by the Chilean version of the EATQ-R and indicators of adolescents' adjustment and maladjustment, such as self-esteem, self-efficacy, depressive symptoms, behavioral problems, and substance use. These findings support our hypotheses. The temperamental factor Effortful Control displayed a significant inverse association with indicators of adolescent maladjustment (depressive symptoms, behavioral problems, and substance use). In addition, it was directly associated with adolescent self-esteem and self-efficacy. In contrast, the opposite pattern was observed with respect to the Negative Affect dimension. This temperamental dimension was inversely related to indicators of adolescent self-concept and directly related to indicators of adolescent maladjustment.

These results are in line with those of previous studies, which indicate that having poor self-regulation skills is related to internalizing and externalizing symptomatology (Nigg, 2006; Griffith et al., 2010; Baetens et al., 2011; Muris et al., 2011), poor academic performance (Valiente et al., 2013), difficulties emotionally and behaviorally adapting to the social demands of a classroom environment (Al-Hendawi, 2013), and problematic peers interaction, such aggression and peer-victimization (Coplan and Bullock, 2012).

Additionally, Nigg (2006) indicates that high effortful control protects against a wide range of psychopathology. Based on cross-sectional and longitudinal studies, it has been demonstrated that low effortful control is related to externalizing disorders (Eisenberg et al., 2005, 2016; Olson et al., 2017).

On the other hand, temperamental negative emotionality or irritability tends to predict both internalizing and externalizing problems. Negative emotionality seems to be on the base of comorbidity of internalizing and externalizing disorders (Keiley et al., 2003). Mikolajewski et al. (2013) have shown that negative affect shares genetic and environmental influences with both internalizing and externalizing disorders in childhood. Rothbart and Posner (2006) differentiate between fear-related affect vs. anger and irritability, explaining that that the former is particularly related to internalizing disorders, and the latter to externalizing disorders. Psychopathology has also been found to be closely linked to negative emotionality (Tackett et al., 2013; Snyder et al., 2015).

Additionally, negative emotions appear to interact with effortful control in predicting internalizing and externalizing behavioral problems. For example, internalizing problems were shown to be related to negative emotionality and high reactive control (Eisenberg et al., 2005). Moreover, anxiety disorders appear to result from the confluence of high negative emotionality and low effortful control (Lonigan et al., 2004). High negative emotionality and low effortful control are related to maladaptive cognitive process, deficits in emotion regulation, and subsequent adolescent psychopathology (Snyder et al., 2015).

In the present study, both indicators of adolescent adjustment (self-esteem and self-efficacy) were related to effortful control and negative affect, with the former being a protective factor and the latter a risk factor for adolescent development. These associations were clearly established, even more so than in past studies (Heinonen et al., 2002; Robins et al., 2010).

We did not hypothesize on Affiliativeness and Surgency and their association with indicators of adolescent adjustment. Nevertheless, based on the study results, Affiliativeness could be considered as protective factor (similar to Effortful Control).

Affiliativeness was positively related to indicators of adolescent adjustment (self-esteem and self-efficacy) and negatively related with antisocial behavior. Affiliativeness reflects socialization tendencies that are related to positive emotions (Graziano and Tobin, 2017). Nevertheless, most consistent evidence links it with effortful control in children (Eisenberg et al., 2016) and adults (Putnam et al., 2001), not in adolescents.

The positive relation between Affiliativeness and Depressive Mood could be an indicator of the ability to feel empathy, which implies the capacity to connect with one's own negative emotions. Jensen-Campbell et al. (2015) proposed an association between personality traits (for example, Affiliativeness) and interpersonal relationships. Nigg (2006) proposed that affiliation is rooted, at least in later development (in adolescence), in the capacity to feel and recognize negative emotions in others (like sadness or anxiety), and thus to feel empathy for others.

Surgency was positively related with indicators of adolescent adjustment, and it was negatively related to depressive symptoms. However, at the same time, this temperamental dimension was positively related to antisocial behavior and substance abuse. Nigg (2006) indicated that recent physiological evidence suggests that this temperamental trait is related to motivation (approach) instead of the valance of experiences (positive or negative). Therefore, correlations between Surgency and indicators of adolescent maladjustment could be interpreted as adolescent exploratory behavior, based on their openness to new experiences.

Finally, even though the utilized methodology followed suitable procedures for examining the validity and reliability of a self-report questionnaire, the study has three mayor limitations. Firstly, it lacked divergent validity indicators, and second, the convergent validity study did not consider the activity level dimension. Lastly, the instrument was only applied in adolescents between 12 and 18 years of age, leaving out younger (10–11 years old) early adolescents.

Despite these limitations, it can be concluded that the Chilean version of the EATQ-R questionnaire, with 83 items, is a useful self-report tool for measuring temperament in adolescents between 12 and 18 years of age.

Temperament is an early identifiable risk factor for the development of psychopathology. The period from childhood to adolescence is thus a valuable window in which to identify risk factors that may underpin the development and persistence of common mental disorders (Forbes et al., 2017). In this sense, the EATQ-R could potentially become an effective early detection tool, to intervene in the adjustment difficulties of adolescents in a timely manner in order to prevent later psychopathology and mental health problems.

## Author contributions

MH: Contributions to the conception and design of the work; interpretation of data for the work; Drafting the work and revising it critically for important intellectual content; Final approval of the version to be published. JP: Contributions to the conception, and design of the project; analysis, interpretation of data for the work; Revising it critically for important intellectual content; Final approval of the version to be published. CG: contributions to the conception of the work; Drafting the work; Final approval of the version to be published. GR and VM: Contributions to the conception, of the project; Revising it critically for important intellectual content; Final approval of the version to be published. MH, JP, CG, GR, and VM: Agreement to be accountable for all aspects of the work in ensuring that questions related to the accuracy or integrity of any part of the work are appropriately investigated and resolved.

### Conflict of interest statement

The authors declare that the research was conducted in the absence of any commercial or financial relationships that could be construed as a potential conflict of interest.

## Footnotes

^1^Available online at: https://research.bowdoin.edu/rothbart-temperament-questionnaires/instrument-descriptions/the-early-adolescent-temperament-questionnaire

^2^Institute for Digital Research and Education, UCLA., (s. f.). Retrieved of http://www.statmodel.com/discussion/messages/8/1223.html?1322676286

^3^Given the length of the questionnaires administered in FONDECYT project number 11130041, the following items were not included: 56 (Attention); 73 (Frustration); 72 (Aggression); 94 (Affiliation); 69 (Shyness); 95 and 103 (High Intensity Pleasure); and all items (1; 36; 64; 83; 100) of the Activity Level dimension.

## References

[B1] Al-Hendawi M. (2013). Temperament, school adjustment, and academic achievement: existing research and future directions. Educ. Rev. 65, 177–205. 10.1080/00131911.2011.648371

[B2] Baetens I. Claes L. Willem L. Muehlenkamp J. Bijttebier P. (2011). The relationship between non-suicidal self-injury and temperament in male and female adolescents based on child- and parent-report. Pers. Indivd. Differ. 50, 527–530. 10.1016/j.paid.2010.11.015

[B3] Bentler P. M. (1990). Comparative fit indexes in structural models. Psychol. Bull. 107, 238–246. 10.1037/0033-2909.107.2.238 2320703

[B4] Bowen A. M. Kromschroder M. Martínez M. J. Lecannelier F. (2004). Adaptación y Validación del Instrumento de Temperamento: Cuestionario Para Evaluar el Comportamiento en la Infancia. [Adaptation and Validation of the Temperament Instrument: Early Childhood Behavior Questionnaire]. Graduate Theses and Dissertations, Universidad del Desarrollo, Santiago.

[B5] Browne M. W. Cudeck R. (1993). Alternative ways of assessing model fit, in Testing Structural Equation Models, eds Bollen K. A. Long J. S. (Beverly Hills, CA: Sage), 136–162.

[B6] Brumley L. D. Jaffee S. R. (2016). Defining and distinguishing promotive and protective effects for childhood externalizing psychopathology: a systematic review. Soc. Psychiatry Psychiatr. Epidemiol. 51, 803–815. 10.1007/s00127-016-1228-1 27130443

[B7] Byrne B. M. (1998). Structural Equation Modeling with LISREL, PRELIS and SIMPLIS: Basic Concepts, Applications and Programming. Mahwah, NJ: Lawrence Erlbaum Associates.

[B8] Calkins S. D. Perry N. Dollar J. (2016). A biopsychosocial model of self-regulation in infancy, in Child Psychology. A Handbook of Contemporary Issues, 3rd Edn, eds Balter L. Tamis-LeMonda C. S. (New York, NY: Routledge), 3–20.

[B9] Chang C. (2004). Temperament in Chinese Children: A Comparison of Gender and self/Parental Ratings. Doctoral Theses, University of Hawaii, Honolulu, HI.

[B10] Coplan R. J. Bullock A. (2012). Temperament and peer relationships, in Handbook of Temperament, eds Zentner M. Shiner R. L. (New York, NY: Guilford Press), 442–461.

[B11] Cumsille P. Martinez M. L. Rodriguez V. Darling N. (2014). Análisis psicométrico de la Escala Parental Breve (EPB): invarianza demográfica y longitudinal en adolescentes chilenos. [Psychometric analysis of the Escala Parental Breve (EPB): demographic invariance in chilean adolescents]. Psykhe 23, 1–14. 10.7764/psykhe.23.2.665

[B12] De Pauw S. Mervielde I. (2010). Temperament, personality and developmental psychopathology: a review based on the conceptual dimensions underlying childhood traits. Child Psychiat. Hum. D. 41, 313–329. 10.1007/s10578-009-0171-8 20238477

[B13] Diamantopoulos A. Siguaw J. A. (2000). Introducing LISREL. London: Sage Publications.

[B14] Ding L. Velicer W. F. Harlow L. L. (1995). Effects of estimation methods, number of indicators per factor, and improper solutions on structural equation modeling fit indices. Struct. Equ. Model. 119–143. 10.1080/10705519509540000

[B15] Eisenberg N. Sadovsky A. Spinrad T. Fabes R. A. Losoya S. Valiente C.. (2005). The relations of problem behavior status to children's negative emotionality, effortful control, and impulsivity: Concurrent relations and prediction of change. Dev. Psychol. 41, 193–211. 10.1037/0012-1649.41.1.193 15656749 PMC1361290

[B16] Eisenberg N. Spinrad T. Valiente C. (2016). Emotion-related self-regulation, and children's social, psychological, and academic functioning, in Child Psychology. A Handbook of Contemporary Issues, 3th^.^ Edn, eds Balter L. Tamis-LeMonda C. S. (New York, NY: Routledge), 219–244.

[B17] Ellis L. (2002). Individual Differences and Adolescent Psychosocial Development. Doctoral Thesis, University of Oregon, Eugene, OR.

[B18] Ellis L. K. Rothbart M. K. (2001). Revision of the Early Adolescent Temperament Questionnaire, in Poster presented at the Biennial Meeting of the Society for Research in Child Development (Minneapolis, MN).

[B19] Fan X. Thompson B. Wang L. (1999). Effects of sample size, estimation methods, and model specification on structural equation modeling fit indexes. Struct. Equ. Model. 6, 56–83. 10.1080/10705519909540119

[B20] Findley D. (2013). Self-Concept Clarity and Self-Esteem in Adolescence: Associations with Psychological, Behavioral, and Academic Adjustment. Graduate Theses and Dissertations, University of South Florida, Tampa, FL.

[B21] Forbes M. K. Rapee R. M. Camberis A. L. McMahon C. A. (2017). Unique associations between childhood temperament characteristics and subsequent psychopathology symptom trajectories from childhood to early adolescence. J. Abnorm. Child Psychol. 45, 1221–1233. 10.1007/s10802-016-0236-7 27942995 PMC5466502

[B22] Graziano W. G. Tobin N. (2017). Agreeableness: A dimension of personality, in The Oxford Handbook of the Five Factor Model, ed Widiger T. (New York, NY: Oxford University Press), 105–132.

[B23] Griffith J. W. Zinbarg R. E. Craske M. G. Mineka S. Rose R. D. Waters A. M.. (2010). Neuroticism as a common dimension in the internalizing disorders. Psychol. Med. 40, 1125–1136. 10.1017/S0033291709991449 19903363 PMC2882529

[B24] Hankin B. L. Stone L. Wright P. A. (2010). Corumination, interpersonal stress generation, and internalizing symptoms: accumulating effects and transactional influences in a multiwave study of adolescents. Dev. Psychopathol. 22, 217–235. 10.1017/S0954579409990368 20102657 PMC4031463

[B25] Harter S. (1982). The perceived competence scale for children. Child Dev. 53, 87–97. 10.2307/1129640 6525886

[B26] Heinonen K. Räikkönen K. Keskivaara P. Keltikangas-Järvinen L. (2002). Difficult temperament predicts self-esteem in adolescence. Eur. J. Pers. 16, 439–455. 10.1002/per.464

[B27] Hirvonen R. Väänänen J. Aunola K. Ahonen T. Kiuru N. (2017). Adolescents' and mothers' temperament types and their roles in early adolescents' socioemotional functioning. Int. J. Behav. Dev. [Epub ahead of print]. 10.1177/0165025417729223 PMC6104198 30166742

[B28] Hu L. Bentler P. M. (1999). Cutoff criteria for fit indexes in covariance structure analysis: conventional criteria versus new alternatives. Struct. Equat. Model. 6, 1–55. 10.1080/10705519909540118

[B29] Jackson D. L. (2001). Sample size and number of parameter estimates in maximum likelihood confirmatory factor analysis: a Monte Carlo investigation. Struct. Equat. Model. 8, 205–223. 10.1207/S15328007SEM0802_3

[B30] Jensen-Campbell L. A. Iyer-Eimerbrink P. A. Knack J. M. (2015). Interpersonal traits, in American Psychological Association (APA) Handbooks in Psychology, Handbook of Personality and Social Psychology: Vol. 4. Personality Processes and Individual Differences, eds Mikulincer M. Shaver P. R. (Washington, DC: APA), 351–368.

[B31] Keiley M. K. Lofthouse N. Bates J. E. Dodge K. A. Pettit G. S. (2003). Differential risks of covarying and pure components in mother and teacher reports of externalizing and internalizing behavior across ages 5 to 14. J. Abnorm. Child Psychol. 3, 267–283. 10.1023/A:1023277413027 PMC2764261 12774860

[B32] Kenny D. A. (2012). Measuring Model Fit. Available online at: http://davidakenny.net/cm/fit.htm.

[B33] Kim S. Brody G. McBride V. (2003). Factor structure of the adolescent temperament questionnaire and measurement invariance across gender. J. Early Adolesc. 23, 268–294. 10.1177/0272431603254178

[B34] Lengua L. J. Long A. C. (2002). The role of emotionality and self-regulation in the appraisal coping process: tests of direct and moderating effects. J. Appl. Dev. Psychol. 23, 471–493. 10.1016/S0193-3973(02)00129-6

[B35] Lonigan C. J. Vasey M. W. Phillips B. M. Hazen R. A. (2004). Temperament, anxiety, and the processing of threat-relevant stimuli. J. Clin. Child Adolesc. 33, 8–20. 10.1207/S15374424JCCP3301_2 15028537

[B36] Martel M. M. Pierce L. Nigg J. T. Jester J. M. Adams K. Puttler L. I.. (2009). Temperament pathways to childhood disruptive behavior and adolescent substance abuse: testing a cascade model. J. Abnorm. Child Psychol. 37, 363–373. 10.1007/s10802-008-9269-x 18787942 PMC2659729

[B37] Mikolajewski A. J. Allan N. P. Hart S. Lonigan C. J. Taylor J. (2013). Negative affect shares genetic and environmental influences with symptoms of childhood internalizing and externalizing disorders. J. Abnorm. Child Psychol. 41, 411–423. 10.1007/s10802-012-9681-0 23011215 PMC3548041

[B38] Muris P. Mayer B. Reinders E. Wesenhagen C. (2011). Person-related protective and vulnerability factors of psychopathology symptoms in non-clinical adolescents. Community Ment. Health J. 47, 47–60. 10.1007/s10597-009-9249-9 19816772 PMC3030948

[B39] Muris P. Meesters C. (2009). Reactive and regulative temperament in youths: psychometric evaluation of the Early Adolescent Temperament Questionnaire-Revised. J. Psychopathol. Behav. 31, 7–19. 10.1007/s10862-008-9089-x

[B40] Muris P. Ollendick T. H. (2005). The role of temperament in the etiology of child psychopathology. Clin. Child Fam. Psychol. 8, 271–289. 10.1007/s10567-005-8809-y 16362256

[B41] Muthén L. K. Muthén B. O. (1998-2010). Mplus User's Guide, 6th Edn. Los Angeles, CA: Muthén & Muthén.

[B42] Nigg J. T. (2006). Temperament and developmental psychopathology. J. Child Psychol.Psyc. 47, 395–422. 10.1111/j.1469-7610.2006.01612.x 16492265

[B43] Oldehinkel A. J. Hartman C. A. De Winter A. F. Veenstra R. Ormel J. (2004). Temperament profiles associated with internalizing and externalizing problems in preadolescence. Dev. Psychopathol. 16, 421–440. 10.10170/S0954579404044591 15487604

[B44] Olson S. L. Choe D. E. Sameroff A. (2017). Trajectories of child externalizing problems between ages 3 and 10 years: Contributions of children's early effortful control, theory of mind, and parenting experiences. Dev. Psychopathol. 29, 1333–1351. 10.1017/S095457941700030X 28290269 PMC11227349

[B45] Pérez J. C. Cumsille P. (2012). Adolescent temperament and parental control in the development of the adolescent decision-making in a Chilean sample. J. Adolesc. 35, 659–669. 10.1016/j.adolescence.2011.09.002 21951646

[B46] Putnam S. P. Ellis L. K. Rothbart M. K. (2001). The structure of temperament from infancy through adolescence, in Advances in Research on Temperament, eds Eliasz A. Anglietner A. (Lengerich: Pabst Science Publishers), 164–182.

[B47] Raykov T. (2000). On the large-sample bias, variance, and mean squared error of the conventional noncentrality parameter estimator of covariance structure models. Struct. Equat. Model. 7, 431–441. 10.1207/S15328007SEM0703_4

[B48] Raykov T. (2005). Bias-corrected estimation of noncentrality parameters of covariance structure models. Struct. Equat. Model. 12, 120–129. 10.1207/s15328007sem1201_6

[B49] Robins R. Donnellan B. Widaman K. Conger R. (2010). Evaluating the link between self-esteem and temperament in Mexican origin early adolescents. J. Pers. Soc. Psychol. 83, 185–197. 10.1016/j.adolescence.2009.07.009 PMC2862796 19740537

[B50] Rothbart M. K. (2011). Becoming Who We Are: Temperament, Personality and Development. New York, NY: Guilford Press.

[B51] Rothbart M. K. Bates J. E. (2006). Temperament, in Handbook of Child Psychology: Vol. 3. Social, Emotional, and Personality Development, eds Eisenberg N. Damon W. (New York, NY: Wiley), 99–166.

[B52] Rothbart M. K. Posner M. I. (2006). Temperament, attention, and developmental psychopathology, in Developmental Psychopathology: Developmental Neuroscience, eds Cicchetti D. Cohen D. J. (Hoboken, NJ: John Wiley), 465–501.

[B53] Rothbart M. K. Rueda M. R. (2005). The development of effortful control, in Developing Individuality in the Human Brain: Developing Individuality in the Human Brain: A Tribute to Michael I. Posner, eds Mayr U. Awh E. Keele S. W. (Washington, DC: American Psychological Association), 167–188.

[B54] Satorra A. Bentler P. M. (1994). Corrections for test statistics and standard errors in covariance structure analysis, in Latent Variable Analysis: Applications for Developmental Research, eds Von Eye A. Clogg C. C. (Thousand Oaks, CA: Sage), 399–419.

[B55] Sherer M. Adams C. H. (1983). Construct validation of the Self-Efficacy Scale. Psychol. Rep. 53, 899–902. 10.2466/pr0.1983.53.3.899

[B56] Snyder H. R. Gulley L. Bijttebier P. Hartman C. Oldehinkel A. Mezulis A.. (2015). Adolescent emotionality and effortful control: Core latent constructs and links to psychopathology and functioning. J. Pers. Soc. Psychol. 109, 1132–1149. 10.1037/pspp0000047 26011660 PMC4659771

[B57] Tackett J. L. Lahey B. B. van Hulle C. Waldman I. Krueger R. F. Rathouz P. J. (2013). Common genetic influences on negative emotionality and a general psychopathology factor in childhood and adolescence. J. Abnorm. Psychol. 122, 1142–1153. 10.1037/a0034151 24364617 PMC3938013

[B58] Valiente C. Eisenberg N. Spinrad T. L. Haugen R. Thompson M. S. Kupfer A. (2013). Effortful control and impulsivity as concurrent and longitudinal predictors of academic achievement. J. Early Adolesc. 33, 946–972. 10.1177/0272431613477239

[B59] Viñas F. González M. Gras E. Jane C. Casas F. (2015). Psychometric properties of the EATQ-R among a sample of Catalan-speaking Spanish adolescents. Univ. Psychol. 14, 731–742. 10.11144/Javeriana.upsy14-2.ppe

[B60] Watson D. Suls J. Haig J. (2002). Global self-esteem in relation to structural models of personality and affectivity. J. Pers. Soc. Psychol. 83, 185–197. 10.1037/0022-3514.83.1.185 12088125

