# Supplementary material for: Chilean Adaptation and Validation of the Early Adolescent Temperament Questionnaire-Revised Version
Source: Front Psychol. 2017 Dec 18;8:2131. doi: 10.3389/fpsyg.2017.02131 (PMC5742495; doi:10.3389/fpsyg.2017.02131)
Supplement: Supplementary file 1 [file DataSheet1.docx]

| Appendix 1. Factor Loadings of Confirmatory Factor Analysis | | | | | | | | | | | | | | | |
| --- | --- | --- | --- | --- | --- | --- | --- | --- | --- | --- | --- | --- | --- | --- | --- |
|  | Descriptive statistic | | | | | | | | | | | | | AFC (STDY) ^a^ | |
| Item | *N* | | | *M* | | *DS* | | | | Skewness | | | Kurtosis | Initial Version | Final Version |
| Activation Control | | | | | | | | | | | | | | | |
| EATQR007 (R) | 621 | | | 2,81 | | 1,13 | | | | 0,06 | | | -0,70*** | -0,48*** | -0,48*** |
| EATQR024 (R) | 621 | | | 4,14 | | 1,02 | | | | -1,22*** | | | 1,14*** | -0,30*** | - |
| EATQR032 | 622 | | | 2,80 | | 1,04 | | | | 0,11 | | | -0,31 | 0,38*** | 0,38*** |
| EATQR055(R) | 619 | | | 2,82 | | 1,23 | | | | 0,13 | | | -0,81*** | -0,13 | - |
| EATQR063 | 620 | | | 3,02 | | 1,27 | | | | 0,02 | | | -0,94*** | 0,55*** | 0,57*** |
| EATQR065 | 621 | | | 3,76 | | 1,13 | | | | -0,69*** | | | -0,18 | 0,49*** | 0,50*** |
| EATQR066 | 620 | | | 3,30 | | 1,05 | | | | -0,10 | | | -0,39* | 0,65*** | 0,67*** |
| EATQR082(R) | 621 | | | 3,09 | | 1,23 | | | | -0,12 | | | -0,86*** | -0,51*** | -0,50*** |
| Affiliation | | | | | | | | | | | | | | | |
| EATQR023 | 622 | | | 3,27 | | 1,33 | | | | -0,25* | | | -1,05*** | 0,39*** | 0,37*** |
| EATQR038 | 623 | | | 4,29 | | 0,89 | | | | -1,38*** | | | 1,99*** | 0,49*** | 0,47*** |
| EATQR047 | 618 | | | 3,84 | | 1,25 | | | | -0,86*** | | | -0,31 | 0,55*** | 0,54*** |
| EATQR051 | 619 | | | 4,47 | | 0,80 | | | | -1,80*** | | | 3,76*** | 0,44*** | 0,44*** |
| EATQR075 | 617 | | | 3,84 | | 1,11 | | | | -0,84*** | | | 0,14 | 0,47*** | 0,46*** |
| EATQR078 | 621 | | | 3,28 | | 1,27 | | | | -0,22* | | | -0,93*** | 0,48*** | 0,48*** |
| EATQR088 | 614 | | | 3,86 | | 1,00 | | | | -0,61*** | | | -0,10 | 0,51*** | 0,50*** |
| EATQR094 | 616 | | | 3,00 | | 1,22 | | | | -0,03 | | | -0,84*** | 0,35*** | 0,34*** |
| Attention | | | | | | | | | | | | | | | |
| EATQR025 | 621 | | | 3,08 | | 1,21 | | | | -0,05 | | | -0,84*** | 0,63*** | 0,64*** |
| EATQR044(R) | 621 | | | 3,31 | | 1,12 | | | | -0,20* | | | -0,57*** | -0,40*** | -0,40*** |
| EATQR056(R) | 617 | | | 2,21 | | 1,21 | | | | 0,71 | | | -0,44* | -0,33*** | -0,33*** |
| EATQR062(R) | 621 | | | 3,44 | | 1,29 | | | | -0,39*** | | | -0,90*** | -0,61*** | -0,61*** |
| EATQR067 | 620 | | | 3,58 | | 1,06 | | | | -0,53*** | | | -0,14 | 0,22*** | - |
| EATQR086(R) | 613 | | | 3,02 | | 1,08 | | | | 0,10 | | | -0,44* | -0,54*** | -0,55*** |
| EATQR097 | 618 | | | 3,78 | | 1,02 | | | | -0,69*** | | | 0,18 | 0,51*** | 0,49*** |
| Inhibitory Control | | | | | | | | | | | | | | | |
| EATQR012(R) | 623 | | | 2,97 | | 1,21 | | | | -0,04 | | | -0,87*** | -0,25*** | - |
| EATQR019 | 621 | | | 3,18 | | 1,09 | | | | -0,02 | | | -0,56*** | 0,33*** | 0,31*** |
| EATQR020 | 618 | | | 3,41 | | 1,23 | | | | -0,33*** | | | -0,84*** | 0,44*** | 0,45*** |
| EATQR021(R) | 617 | | | 2,75 | | 1,24 | | | | 0,23** | | | -0,87*** | -0,32*** | - |
| EATQR022(R) | 618 | | | 2,04 | | 1,21 | | | | 1,01*** | | | 0,03 | -0,28*** | - |
| EATQR039(R) | 623 | | | 3,85 | | 1,24 | | | | -0,83 | | | -0,38 | -0,26*** | - |
| EATQR045(R) | 621 | | | 2,22 | | 1,08 | | | | 0,58*** | | | -0,31 | -0,01 | - |
| EATQR046(R) | 618 | | | 2,76 | | 1,20 | | | | 0,19 | | | -0,79*** | -0,44*** | -0,33*** |
| EATQR071 | 619 | | | 4,10 | | 1,17 | | | | -1,31*** | | | 0,86*** | 0,28*** | - |
| EATQR081 | 618 | | | 3,56 | | 1,13 | | | | -0,48*** | | | -0,41* | 0,53*** | 0,55*** |
| EATQR089 | 616 | | | 3,75 | | 0,99 | | | | -0,56*** | | | -0,01 | 0,55*** | 0,64*** |
| Frustration | | | | | | | | | | | | | | | |
| EATQR040(R) | 621 | | | 3,20 | | 1,24 | | | | 0,16 | | | -0,81*** | -0,44*** | -0,41*** |
| EATQR042 | 622 | | | 2,63 | | 1,34 | | | | 0,31*** | | | -1,05*** | 0,49*** | 0,48*** |
| EATQR058 | 619 | | | 3,48 | | 1,15 | | | | -0,28** | | | -0,71*** | 0,62*** | 0,61*** |
| EATQR073 | 620 | | | 3,30 | | 1,20 | | | | -0,19 | | | -0,77*** | 0,38*** | 0,38*** |
| EATQR079 | 620 | | | 3,81 | | 1,08 | | | | -0,60*** | | | -0,43* | 0,54*** | 0,54*** |
| EATQR091 | 614 | | | 3,45 | | 1,25 | | | | -0,37*** | | | -0,86*** | 0,48** | 0,47*** |
| EATQR098 | 616 | | | 3,08 | | 1,24 | | | | 0,02 | | | -0,93*** | 0,40*** | 0,40*** |
| EATQR101 | 610 | | | 3,41 | | 1,12 | | | | -0,25* | | | -0,59*** | 0,46*** | 0,46*** |
| EATQR102 | 615 | | | 3,18 | | 1,21 | | | | -0,06 | | | -0,86*** | 0,51*** | 0,53*** |
| Activity Level | | | | | | | | | | | | | | | |
| EATQR001 | 621 | | | | 3,39 | | 1,26 | | -,32*** | | -,84*** | | | 0,77*** | 0,76*** |
| EATQR016 | 620 | | | | 2,95 | | 1,12 | | 0,16 | | -0,53*** | | | 0,26*** | - |
| EATQR036 | 623 | | | | 3,72 | | 1,25 | | -0,67*** | | -0,59*** | | | 0,75*** | 0,75*** |
| EATQR064 | 621 | | | | 3,41 | | 1,33 | | -0,38*** | | -1,01*** | | | 0,62*** | 0,62*** |
| EATQR083 | 621 | | | | 2,92 | | 1,37 | | 0,08 | | -1,18*** | | | 0,62*** | 0,65*** |
| EATQR0100 | 616 | | | | 4,08 | | 1,09 | | -1,09*** | | 0,54*** | | | 0,47*** | 0,46*** |
| Pleasure Sensitivity | | | | | | | | | | | | | | | |
| EATQR028 | 618 | | 2,99 | | | | 1,17 | | 0,00 | | -0,61*** | | | 0,41*** | 0,40*** |
| EATQR030 | 620 | | 3,94 | | | | 1,21 | | -0,96*** | | -0,09 | | | 0,63*** | 0,65*** |
| EATQR034 | 621 | | 3,90 | | | | 1,13 | | -0,84*** | | -0,07 | | | 0,27*** | - |
| EATQR048 | 621 | | 3,86 | | | | 1,31 | | -0,89** | | -0,41* | | | 0,63*** | 0,63*** |
| EATQR050 | 617 | | 3,58 | | | | 1,37 | | -0,56*** | | -0,91*** | | | 0,48*** | 0,46*** |
| EATQR070 | 621 | | 3,48 | | | | 1,31 | | -0,44*** | | -0,91*** | | | 0,75*** | 0,77*** |
| EATQR080 | 620 | | 3,82 | | | | 1,24 | | -0,80*** | | -0,36 | | | 0,66*** | 0,66*** |
| Perceptual Sensitivity | | | | | | | | | | | | | | | |
| EATQR004 | 621 | | 3,36 | | | | 1,39 | | -0,39*** | | -1,10*** | | | 0,48*** | 0,48*** |
| EATQR011 | 621 | | 3,38 | | | | 1,36 | | -0,37*** | | -1,03*** | | | 0,66*** | 0,68*** |
| EATQR031 | 621 | | 3,65 | | | | 1,20 | | -0,61*** | | -0,53*** | | | 0,74*** | 0,76*** |
| EATQR035 | 620 | | 3,80 | | | | 1,12 | | -0,75*** | | -0,13 | | | 0,61*** | 0,61*** |
| EATQR041 | 623 | | 4,15 | | | | 0,97 | | -1,31*** | | 1,68*** | | | 0,31*** | - |
| EATQR092(R) | 610 | | 2,37 | | | | 1,35 | | 0,59*** | | -0,85*** | | | -0,16*** | - |
| Shyness | | | | | | | | | | | | | | | |
| EATQR006 | 622 | | 2,25 | | | | 1,24 | | 0,65*** | | -0,62*** | | | 0,57*** | 0,56*** |
| EATQR009 | 619 | | 2,12 | | | | 1,11 | | 0,69*** | | -0,33 | | | 0,59*** | 0,58*** |
| EATQR043 | 623 | | 2,25 | | | | 1,27 | | 0,62*** | | -0,71*** | | | 0,47*** | 0,46*** |
| EATQR052(R) | 617 | | 3,57 | | | | 1,19 | | -0,54*** | | -0,55*** | | | -0,45*** | -0,45*** |
| EATQR069 | 619 | | 3,89 | | | | 1,16 | | -0,84*** | | -0,13 | | | 0,32*** | 0,33*** |
| EATQR076 | 618 | | 2,54 | | | | 1,27 | | 0,36*** | | -0,90*** | | | 0,84*** | 0,84*** |
| EATQR087(R) | 614 | | 3,22 | | | | 1,44 | | -0,17 | | -1,30*** | | | -0,75*** | -0,76*** |
| High Intensity Pleasure | | | | | | | | | | | | | | | |
| EATQR002 | 622 | 3,01 | | | | 1,23 | | -0,04 | | | | -0,81*** | | 0,11*** | - |
| EATQR008 | 618 | 2,28 | | | | 1,24 | | 0,69*** | | | | -0,56*** | | 0,12*** | - |
| EATQR014(R) | 623 | 2,80 | | | | 1,38 | | 0,12 | | | | -1,20*** | | -0,52*** | -0,55*** |
| EATQR017(R) | 623 | 2,67 | | | | 1,39 | | 0,26** | | | | -1,19*** | | -0,54*** | -0,57*** |
| EATQR027 | 621 | 4,10 | | | | 1,09 | | -1,11*** | | | | 0,48** | | 0,21*** | - |
| EATQR029 | 617 | 3,32 | | | | 1,41 | | -0,34*** | | | | -1,16*** | | 0,19*** | - |
| EATQR053 | 621 | 3,46 | | | | 1,44 | | -0,42*** | | | | -1,20*** | | 0,69*** | 0,68*** |
| EATQR061 | 618 | 3,48 | | | | 1,46 | | -0,47*** | | | | -1,19*** | | 0,54*** | 0,52*** |
| EATQR095 | 617 | 3,89 | | | | 1,26 | | -0,91*** | | | | -0,26 | | 0,37*** | 0,35*** |
| EATQR099 | 612 | 3,54 | | | | 1,39 | | -0,53*** | | | | -0,98*** | | 0,55*** | 0,54*** |
| EATQR103 | 618 | 3,65 | | | | 1,12 | | -0,41*** | | | | -0,51*** | | 0,38*** | 0,33*** |
| Fear | | | | | | | | | | | | | | | |
| EATQR003 | 622 | 3,64 | | | | 1,26 | | -0,66*** | | | | -0,53*** | | 0,45*** | 0,42*** |
| EATQR054 | 619 | 2,35 | | | | 1,30 | | 0,55*** | | | | -0,86*** | | 0,29*** | - |
| EATQR057 | 618 | 3,83 | | | | 1,14 | | -0,76*** | | | | -0,18 | | 0,34*** | 0,36*** |
| EATQR077 | 619 | 2,37 | | | | 1,30 | | 0,59*** | | | | -0,77*** | | 0,41*** | 0,42*** |
| EATQR085 | 618 | 3,99 | | | | 1,21 | | -1,06*** | | | | 0,13 | | 0,43*** | 0,43*** |
| EATQR093 | 615 | 2,44 | | | | 1,38 | | 0,56*** | | | | -0,97*** | | 0,33*** | 0,32*** |
| Aggresion | | | | | | | | | | | | | | | |
| EATQR005 | 622 | 2,77 | | | | 1,24 | | 0,23** | | | | -0,83*** | | 0,47*** | 0,46*** |
| EATQR010 | 620 | 1,90 | | | | 1,21 | | 1,18*** | | | | 0,27 | | 0,43*** | 0,43*** |
| EATQR018 | 622 | 2,41 | | | | 1,29 | | 0,49*** | | | | -0,85*** | | 0,63*** | 0,65*** |
| EATQR033 | 622 | 2,95 | | | | 1,38 | | 0,08 | | | | -1,19*** | | 0,41*** | 0,42*** |
| EATQR037 | 621 | 2,21 | | | | 1,20 | | 0,83*** | | | | -0,20 | | 0,54*** | 0,53*** |
| EATQR059 | 619 | 2,60 | | | | 1,40 | | 0,36*** | | | | -1,14*** | | 0,49*** | 0,49*** |
| EATQR068 | 620 | 2,14 | | | | 1,01 | | 0,74*** | | | | 0,21 | | 0,29*** | - |
| EATQR072 | 618 | 2,69 | | | | 1,18 | | 0,38*** | | | | -0,62*** | | 0,37*** | 0,36*** |
| EATQR074(R) | 621 | 2,81 | | | | 1,07 | | 0,23** | | | | -0,25 | | -0,20*** | - |
| EATQR084 | 618 | 2,39 | | | | 1,11 | | 0,51*** | | | | -0,41** | | 0,55*** | 0,56*** |
| EATQR096 | 614 | 2,25 | | | | 1,21 | | 0,67*** | | | | -0,52*** | | 0,43*** | 0,43*** |
| Depressive Mood | | | | | | | | | | | | | | | |
| EATQR013 | 621 | 2,52 | | | | 1,21 | | 0,42*** | | | | -0,66*** | | 0,42*** | 0,42*** |
| EATQR015 | 621 | 2,37 | | | | 1,32 | | 0,53*** | | | | -0,91*** | | 0,64** | 0,64*** |
| EATQR026R | 622 | 3,89 | | | | 0,98 | | -0,67*** | | | | 0,11 | | -0,45*** | -0,46*** |
| EATQR049 | 619 | 3,09 | | | | 1,41 | | -0,13 | | | | -1,28*** | | 0,76*** | 0,76*** |
| EATQR060 | 620 | 3,69 | | | | 1,23 | | -0,66*** | | | | -0,55*** | | 0,60*** | 0,58*** |
| EATQR090 | 615 | 2,20 | | | | 1,27 | | 0,81*** | | | | -0,40** | | 0,62*** | 0,61*** |
| *Note.* ^a^ *N*= 503 (listwise). | | | | | | | | | | | | | | | |
